# Efficacy and Safety of Rescue Treatment with Plasma Exchange in Patients with Acute Inflammatory Neurological Disorders: A Single Center Experience

**DOI:** 10.3390/neurolint16040056

**Published:** 2024-07-10

**Authors:** Salvatore Iacono, Giuseppe Schirò, Giuseppe Salemi, Elisabetta Scirè, Paolo Aridon, Michele Melfa, Michele Andolina, Gabriele Sorbello, Andrea Calì, Filippo Brighina, Marco D’Amelio, Paolo Ragonese

**Affiliations:** 1 Department of Biomedicine, Neuroscience and Advanced Diagnostics, University of Palermo, 90129 Palermo, Italy; giuseppeschiro1994@gmail.com (G.S.); paolo.aridon@unipa.it (P.A.); michele.melfa@unipa.it (M.M.); michele.andolina@unipa.it (M.A.); gabriele.sorbello@unipa.it (G.S.); andrea.cali@unipa.it (A.C.); filippo.brighina@unipa.it (F.B.); marco.damelio@unipa.it (M.D.); paolo.ragonese@unipa.it (P.R.); 2 Multiple Sclerosis Center, Foundation Institute G. Giglio, Cefalù, 90015 Palermo, Italy; 3 Trasfusional Medicine Unit, University Hospital Policlinico P. Giaccone, 90129 Palermo, Italy; elisabetta.scire@policlinico.pa.it

**Keywords:** plasma exchange, multiple sclerosis, myasthenia gravis, CIDP, intravenous immunoglobulin, NMOSD, effectiveness, adverse events, rescue therapy, pathogenetic antibodies

## Abstract

Background: Therapeutic plasma exchange (TPE) is a highly effective rescue treatment for patients with acute exacerbation of neuroimmunological disease that removes circulating autoantibodies and inflammatory components from the bloodstream. The aims of this study are to explore the safety and the effectiveness of TPE in patients with autoimmune neurological disorders. Methods: We retrospectively evaluated the frequency of adverse events (AEs) and the effectiveness of TPE using the modified Ranking Scale (mRS) in patients with acute neurological flares who underwent TPE at the University Hospital of Palermo. Results: Of 59 patients, the majority underwent TPE due to multiple sclerosis (MS) relapse. In 23.7% of cases, TPE was performed before obtaining a definite diagnosis due to the severity of the clinical presentation. After TPE, the mRS score was globally reduced (*p* < 0.0001), and this effect was marked in patients with MS, Guillain–Barré syndrome, and myasthenia gravis crisis but not in those with paraneoplastic syndromes. Circulating pathogenetic antibodies, younger age, and the early use of TPE were factors strongly associated with TPE effectiveness. The overall safety profile of TPE was satisfactory with an AE frequency of 15%. Conclusions: These results highlight the early use of TPE in patients with circulating pathogenetic antibodies as well as its favorable safety profile.

## 1. Introduction

Therapeutic plasma exchange (TPE) is a highly effective rescue treatment for immune-mediated disorders of the peripheral nervous system and the central nervous system (CNS) with various degrees of efficacy [1]. TPE consists of removing a given volume of plasma and separate it from corpuscular blood constituents with the subsequent replacement of the removed plasma volume [1]. This procedure allows the removal of the pro-inflammatory cytokines, complement components, and circulating antibodies that are present in a rapid immunomodulatory effect [2,3]. Thus, the obvious rationale of TPE is the removal of pathogenetic circulating autoantibodies and other inflammatory components; however, it remains a nonspecific therapeutic approach [4]. Accordingly, TPE is widely used as first- or second-line rescue therapy in patients with neurological autoimmune or immune-mediated disorders such as multiple sclerosis (MS), neuromyelitis optica spectrum disorder (NMOSD), myasthenia gravis (MG) crisis, Guillain–Barré syndrome (GBS), chronic inflammatory demyelinating neuropathy (CIDP), and others [5]. Despite the recent publication of the ninth edition of guidelines on the use of TPE in clinical practice, there is no evidence supporting the use of TPE instead of corticosteroids or intravenous immunoglobulin (IVIg) as the first-line therapy for many immune-mediated diseases, and the treatment choice is often made on a case-by-case basis [5,6]. For instance, in the case of MG crisis and GBS, IVIg and TPE are used to counteract acute disease exacerbations; however, the choice of appropriate treatment is still debated. Several studies explored the effectiveness of TPE and IVIg in the case of GBS; however, a clear superiority of one therapy over the other has not emerged. Based on the first randomized trial, IVIg and TPE appear to have a similar effectiveness [7]. These studies, however, included predominantly patients with the demyelinating form of GBS [8]. Recently, some authors reported that TPE was more effective in patients with the axonal form of GBS [9]. Conversely, it seems that treatment with IVIg is less commonly associated with the occurrence of adverse events (AEs) when compared to TPE [10,11]. It is also well known that corticosteroids have no effect on GBS course [12]. Regarding the treatment of MG crisis, corticosteroids may cause transient worsening of myasthenic crisis; therefore, the immediate and initial treatment with IVIg or TPE is indicated. However, also in this case, data from comparison studies were not meaningful. Indeed, Mandawat et al. found that the effectiveness of IVIg and TPE were similar in MG crisis; however, therapy with IVIg was cheaper and associated with fewer adverse events (AEs), making it preferable in patients with comorbidity [13]. Other authors confirmed these data and suggested that the choice between IVIg and TPE should be based upon the availability of resources [14]. Finally, TPE also appeared highly effective in patients with MS relapses even if circulating antibodies probably have a minor pathogenetic role in this case. However, the first-line therapy in cases of MS, NMOSD, and myelin oligodendrocyte glycoprotein antibody-associated disease (MOGAD) attacks remain high-dose intravenous methylprednisolone, while TPE or IVIg are employed in cases of steroid-refractory attacks [15]. CIDP deserves a brief mention. In this case, corticosteroids and IVIg are employed as maintenance therapy, while TPE is commonly used in refractory patients [16]. Thus, both IVIg and TPE, by removing the pathogenetic antibodies and immune mediators from systemic circulation, seem to have similar efficacy in neuroimmunological disorders. However, the choice between these two approaches is often tailored to the patient according to some features such as the presence of concomitant infections, comorbidity, the availability of TPE equipment and trained medical personnel, or the cost in the local setting [8]. Nevertheless, since AEs related to TPE (e.g., difficulty with venous access, blood coagulation factor disorder, hypocalcemia, hemodynamic changes, etc.) are not uncommon, they often make the IVIg treatment preferable [13].

In this confusing scenario, IVIg and corticosteroids are preferred in the use of TPE as the first-line rescue therapy during acute exacerbation of neuroimmunological diseases, while its use is usually empirically reserved in the case of severe neurological attacks. Also, in this case, TPE is commonly employed in clinical practice before performing the definite diagnosis resulting in a lower than expected TPE effectiveness. Hence, considering the mechanism of action of TPE and its debated employment in clinical practice, in this study, we evaluated the safety and the effectiveness of treatment with TPE and predictors of efficacy in patients with acute exacerbation of neuroimmunological diseases.

## 2. Materials and Methods

### 2.1. Objective

This study sought to evaluate the safety and effectiveness of TPE in patients affected by autoimmune or immune-mediated neurological disorders.

### 2.2. Study Design

This retrospective cohort study was carried out by including individuals who underwent TPE as planned therapy in the setting of hospital admission (HA) from July 2011 to July 2022, at the Neurology Unit of the University Hospital Policlinico Paolo Giaccone, Palermo, Italy. The study inclusion criteria were as follows: (1) patient has an autoimmune or immune-mediated disease of the central or peripheral nervous system; (2) patient underwent at least one TPE session. All patients with missing data were excluded from this study. 

### 2.3. Subjects

The final diagnoses of patients included in this study were made according to the disease-specific and currently used diagnostic criteria. Particularly, MS, NMOSD, and MOGAD diagnoses were carried out according the 2017 McDonald criteria, International Panel for NMO diagnosis, and International MOGAD Panel criteria, respectively [17,18,19]. MG diagnosis was performed based on the finding of a decreasing U-shaped response at 3 Hz repetitive nerve stimulation and/or increased jitter with single-fiber electromyography and positive testing for autoantibodies against AChRs, MuSK, or LRP4 [20]. GBS and CIDP were recognized according to the Asbury and European Federation of Neurological Societies/Peripheral Nerve Society Guideline, respectively [21,22]. The diagnosis of post-infectious acute disseminated encephalomyelitis (ADEM) was made according to the Bradford Hill causality criteria as well as the identification of a temporal relationship between infectious disease and acute myelitis/EM with typical clinical findings and without other positive serological testing [23]. The diagnosis of paraneoplastic syndromes (PNSs) was made based on the presence of circulating onconeural antibodies and the related neurological manifestation with or without tumor [24]. The patients with SPS had a higher serum titer of anti-GAD 65 IgG (>20 mmol/L) as well as the typical neurological manifestations such as hypertonia, rigidity, and exaggerated startle reaction; therefore, the definite diagnosis of SPS was made [25].

### 2.4. Data Collection

Clinical and demographic data of patients who underwent TPE were reviewed retrospectively by examining the medical records. We collected the reason for performing TPE, final diagnosis, testing results for serum antibodies, and the frequency and severity of AEs that occurred during TPE. We also collected the modified Ranking Scale (mRS) score before and after TPE for all the participants. mRS was scored as follows: 0, no symptoms; 1, no significant disability; 2, slight disability; 3, moderate disability; 4, moderately severe disability; 5, severe disability; and 6, death [26]. Specifically, the Expanded Disability Status Scale (EDSS) was obtained before and after TPE in patients with MS, the Myasthenia Gravis Activity Day Living (MGADL) score was obtained before and after TPE in patients with MG, and the Muscle Research Council (MRC) sum score was obtained before and after TPE in patients with GBS and CIDP [27,28,29]. 

### 2.5. Plasma Exchange

The procedural indication for TPE was addressed by the treating neurologists. TPE was performed by transfusion medicine physicians at the apheresis center according to current guidelines [1]. Citrate anticoagulation was used to prevent clotting of extracorporeal whole blood. During the procedure, patients were regularly assessed for signs and symptoms of hemodynamic instability by regularly examining blood pressure, heart rate, and capillary oxygen saturation. Patients received three to five cycles of TPE per hospital admission (HA) with an interval of one to three days between cycles, according to the transfusion medicine physician’s evaluation. In each cycle, 1000 to 1500 mL of plasma volume was exchanged and replaced with 5% human albumin and electrolytes solution. Complications and adverse reactions during and after the procedure were thoroughly assessed by on-duty neurologists and transfusion medicine physicians.

### 2.6. Defining Effectiveness 

In all the study participants, a one-point reduction in the mRS score represented an improvement, while patients with an unchanged or increased mRS score after TPE were considered as non-responders [30]. Specifically, a reduction of at least two points of the MGADL scale was considered an improvement in MG subjects [31,32]. In patients with GBS and CIDP responding to TPE, the MRC sum score was ameliorated afterwards, whereas non-responders showed no improvement in or even a worsening of the MRC sum score [29]. In MS patients, a reduction of at least 0.5 point in the EDSS was considered a successful response to TPE. In other cases, the mRS score was the only study outcome measure. Thus, TPE effectiveness was defined as follows: (1) a one-point reduction in the mRS score; (2) a 0.5-point reduction in the EDSS score; (3) a two-point reduction in the MGADL score; and (4) any improvement in the MRC sum score.

### 2.7. Defining Adverse Events

An adverse event (AE) was any side effect that occurred during TPE, directly or indirectly related to the treatment. AE severity was established according to the 5th version of the Common Terminology Criteria for AEs: (1) mild, asymptomatic symptoms do not require medical therapy or any medical procedure; (2) moderate events are characterized as symptoms that require non-invasive medical treatment; (3) severe events are characterized as a non-life-threatening condition but requires specific treatment and may require or prolong the hospitalization; (4) life-threatening conditions require intensive care unit admission; (5) death related to AE [33].

### 2.8. Statistical Analysis

Continuous variables were reported as means with standard deviations (SDs) or medians with the interquartile range (IQR) and analyzed using Mann and Whitney or Kruskal–Wallis tests as appropriate. Categorical variables were reported as percentages and analyzed using Chi-squared analysis or Fisher’s exact test, as appropriate. TPE effectiveness was assessed by comparing the mRS, EDSS, MGADL, and MRC sum scores before and after performing TPE using the Wilcoxon signed rank test. To explore factors associated with TPE effectiveness as well as with the occurrence of AEs during TPE, an unconditional logistic regression analysis was performed for each study variable. The odds ratios (ORs) with 95% confidence intervals (CIs) and *p* value (two-tailed test, α = 0.05) were calculated. Multivariate analysis was performed to investigate the independent effect of predictors after adjustment for one or several other factors or to adjust for confounding variables. Parameters associated with the outcome in the univariate analysis with a threshold of *p* = 0.10 were included in the multivariate model. Age and sex were considered as possible a priori confounders, regardless of the level of significance. The model was manually constructed using the likelihood ratio test to compare the log-likelihood of the model with and without a specific variable. For all the analyses, the level of statistical significance was set as a two-sided *p* value less than 0.05. Statistical analyses were performed using the Statistical Package for the Social Sciences software (IBM SPSS Statistics, Version 26.0; 2019. Armonk, NY, USA: IBM Corp).

## 3. Results

All the continuous variables had a non-normal distribution (*p* < 0.05). A total of 59 participants were included in this study for a total of 447 TPE sessions. In most cases, TPE was performed in patients with a definite diagnosis (76.3%), and these diagnoses were predominantly represented by an MS attack followed MG crisis and GBS as shown in Table 1. Conversely, 23.7% of individuals underwent TPE before obtaining a definite diagnosis, and these patients mainly presented with acute myelitis or encephalomyelitis as reported in Table 1.

After diagnostic work-up, patients with unknown diagnosis were subsequently diagnosed with MS (*n* = 5/14; 35.7%), seronegative NMOSD (*n* = 1/14; 7.1%), ADEM (*n* = 3/14; 17.6%), CIDP (*n* = 2/14; 14.3%), and PNSs (*n* = 3/14; 17.6%). 

Finally, out of *n* = 59 individuals included in this study, *n* = 25 (42.4%) patients underwent TPE due to MS relapses, *n* = 9 (15.3%) had MG crisis, *n* = 9 (15.3%) had GBS, *n* = 5 (8.5%) had CIDP, *n* = 5 (8.5%) had PNSs, *n* = 3 (5.1%) had ADEM, *n* = 1 (1.7%) had seronegative NMOSD, *n* = 1 (1.7%) had MOGAD, and *n* = 1 (1.7%) had SPS. Among patients with PNSs, one patient had polyneuropathy, organomegaly, endocrinopathy, M-protein, and skin changes syndrome (POEMS); two patients had cerebellar degeneration associated with Hu and Yo antibodies; one patient had seronegative paraneoplastic neuropathy, and one patient had anti-titin-associated myasthenic syndrome. 

### 3.1. General Features

Women were prevalent in our cohort (*n* = 36; 61%). Globally, TPE was started at a median age of 52 years (41–65) with a median latency of 26 days (14–60) from acute neurological flare. The complete demographic and clinical features of study participants are reported in Table 2.

A higher proportion of male patients was found among those with CIDP and GBS, but this was not statistically significant (*p* for trend = 0.08; Table 3). Patients with MS and NMOSD/MOGAD started the first TPE session at a lower age compared to other patients (*p* < 0.0001; Table 3). Overall, the time in days between acute neurological symptom and TPE initiation was higher in patients with MS and CIDP; however, this did not reach the statistical significance (*p* = 0.09; Table 3). No other statistically significant differences emerged (Table 3).

**Figure 1:**
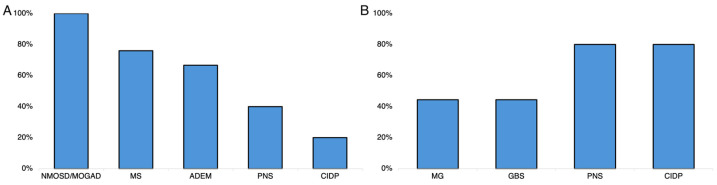
Frequencies of high doses of intravenous methylprednisolone administration (**A**) and intravenous immunoglobulin (**B**) before plasma exchange in patients included in this study. MS, multiple sclerosis; GBS, Guillain–Barré syndrome; CIDP, chronic inflammatory demyelinating polyneuropathy; PNS, paraneoplastic syndrome; NMOSD, neuromyelitis optica spectrum disorders.

Overall, TPE was used as first-line therapy in 19 subjects (32.2%) while *n* = 26 (44.1%) and *n* = 16 (27.1%) individuals received HDIVMP and IVIg before TPE, respectively. The median time between the last HDIVMP administration and TPE initiation was 15.5 (11–44) days, while the last IVIg administration occurred 41.5 days (23–103) before performing TPE. A higher percentage of patients with MS, NMOSD/MOGAD, and ADEM received HDIVMP before starting TPE, while it was not employed in patients with MG and GBS as shown in Figure 1A (*p* for trend <0.0001).

Treatment with IVIg was largely employed before TPE in patients with PNS (*n* = 4; 80%) and CIDP (*n* = 4; 80%). In contrast, it was less used in those with MG (*n* = 4; 44.5%) and GBS (*n* = 4; 44.5%), and other patients did not receive IVIg (*p* for trend <0.0001) as shown in Figure 1B. The patient with SPS did not undergo other therapies before TPE. There were no statistically significant differences regarding the employment of TPE as first-line therapy according to underlying disease (*p* for trend = 0.33) or obtaining a definite diagnosis before performing TPE (35.6% and 21.4%, *p* = 0.5).

### 3.2. Effectiveness Analyses

**Figure 2:**
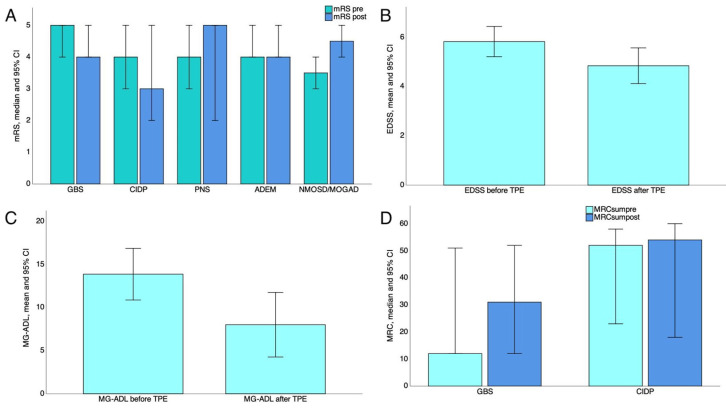
Bar charts showing the comparison before and after plasma exchange of the modified Rankin scale in patients with GBS, CIDP, PNS, ADEM, and NMOSD/MOGAD (**A**); Expanded Disability Status Scale in patients with MS (**B**); MG-ADL score in patients with MG (**C**); MRC sum score in patients with GBS and CIDP (**D**). EDSS, Expanded Disability Status Scale; MG-ADL, Myasthenia Gravis Activity Day Living; GBS, Guillain–Barré syndrome; CIDP, chronic inflammatory demyelinating polyneuropathy; PNS, paraneoplastic syndrome; ADEM, acute disseminated encephalomyelitis; NMOSD, neuromyelitis optica spectrum disorder; MOGAD, myelin oligodendrocyte glycoprotein antibody-associated disease.

Overall, TPE was effective in 43 patients (72.9%) ranging from 40% (*n* = 2/5) in patients with PNS, 66.7% (*n* = 6/9) in patients with GBS and ADEM (*n* = 2/3), 80% in those with MS (20/25), and 60% (*n* = 3/5) in CIDP cases to 100% in MG patients. However, TPE did not show a positive effect in NMOSD/MOGAD patients (*p* for trend = 0.033). Globally, we noted a statistically significant reduction in the mRS score after TPE (from 4 ± 0.9 to 3.4 ± 1.25; F = 23.97; *p* < 0.0001). More specifically, the mRS score did not improve in patients with NMOSD/MOGAD (*p* = 0.32), ADEM (*p* = 1), and PNS (*p* = 0.8) as shown in Figure 2A. On the contrary, the mRS score was reduced in patients with MS (*p* < 0.0001), MG (*p* = 0.016), and GBS (*p* = 0.046). In addition, the mRS score was reduced in those with CIDP; however, this did not reach the statistical significance (*p* = 0.1) as shown in Figure 2A. Finally, we documented a one-point reduction in the mRS score in the patient with SPS, making TPE 100% effective in this patient.

We also observed a reduction of the EDSS (*p* < 0.0001; Figure 2B) and MG-ADL score (*p* = 0.009; Figure 2C) in MS and MG patients, respectively. After TPE, the median MRC sum score slightly increased in patients with CIDP (52 [34–55] and 54 (55–58]; *p* = 0.47) and GBS (12 [12–48] and 31 [24–48]; *p* = 0.11); however, statistical significance was not achieved (Figure 2D).

Finally, TPE effectiveness was lower in patients who underwent TPE before receiving a definite diagnosis (50% vs. 80%; *p* = 0.04). Effectiveness reached 88.2% in patients who tested positive for serum antibodies compared to those who were seronegative (66.7%), but this did not reach the statistically significance (*p* = 0.12).

The multivariate analyses revealed that seropositivity for circulating antibodies was the only factor statistically significant associated with TPE effectiveness; however, age less than 50 years and the use of TPE as first-line therapy were also associated with highest effectiveness (Table 4).

### 3.3. Safety Analyses

**Figure 3:**
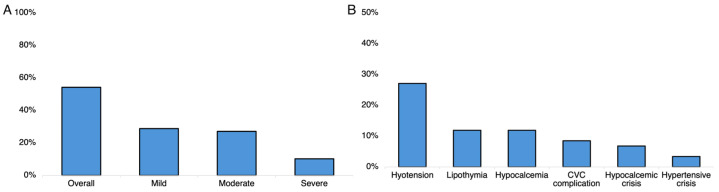
(**A**) Bar charts showing the frequency of adverse events after plasma exchange in study participants according to the grade of severity and the relative frequency of the adverse events per type (**B**). Abbreviation: CVC, central venous catheter.

A total of 32 participants (54.2%) complained at least one AE. In the majority of cases, these were mild (28.8%), while only six participants reported at least one severe AE (10.1%) as shown in Figure 3A. A total number of 66 AEs were documented with an overall frequency of 14.8% over 447 procedures. Out of 66 AEs, *n* = 31 (47%) were mild, *n* = 26 (39.4%) were moderate, and *n* = 9 (13.6%) were severe. A total of *n* = 16 participants reported at least one episode of hypotension (27.2%), which was the most common reported AEs as shown in Figure 3B. The overall AE incidence rate was overall 1.46 per 10 TPE sessions (95% CI 1–2.1). The following values for each AE class were obtained: mild = 0.7 (95% CI 0.5–1.1), moderate = 0.54 (95% CI 0.3–0.9), and severe = 0.19 (95% CI 0.09–0.4). Out of nine severe AEs, *n* = 4 (44.4%) were hypocalcemia crisis, *n* = 3 (33.3%) hypotension, *n* = 1 (11.1%) hypertension and *n* = 1 (11.1%) CVC complication. No life-threatening AEs as well as no death occurred in this study.

The AE frequency did not differ across disease groups (*p* for trend = 0.57), sex (*p* = 0.79), age class (*p* = 0.54), positive serum antibody testing (*p* = 0.48), or use of TPE as a first-line rescue therapy (*p* = 0.7). However, the frequency of AEs was higher in patients who underwent at least five TPE sessions (75% vs. 40%; *p* = 0.008). The multivariate analyses displayed that performing at least five TPE sessions was strongly associated with AEs (OR = 5.79; 95% CI 1.67–20.1; *p* = 0.006), while each point increase in baseline the mRS score was associated with a 49% higher risk of developing an AE (OR = 0.51; 95% CI 0.25–1.04; *p* = 0.063) without other associations (Table 5).

## 4. Discussion

In this study, we explored the safety and the effectiveness of therapeutic TPE in patients with acute exacerbations of neuroimmunological diseases including MS, NMOSD/MOGAD, CIDP, GBS, ADEM, PNSs, and SPS. Most patients included in this study underwent TPE due to MS attack; however, in the 23.7% of cases, TPE was performed before obtaining the definite diagnosis (Table 1). Among these patients, 35.7% had subacute or chronic polyneuropathy subsequently diagnosed as CIDP and PNSs, while the remaining patients had an inflammatory CNS disease subsequently diagnosed as MS, seronegative NMOSD, and ADEM. Based on a one-point reduction in the mRS score, TPE was effective in 72.9% of participants, with an increasing effectiveness trend in favor of patients with MG crisis (*p* for trend = 0.033). Accordingly, we observed a significant reduction in the mRS score after TPE (*p* < 0.0001), although there were some differences according to final diagnosis.

### 4.1. TPE in MS

In our cohort, patients with MS were younger compared to others and underwent their first TPE session at lower age compared to other patients; in addition, TPE was run with a higher latency from acute symptom in these patients (Table 3). Moreover, patients with MS more commonly received HDIVMP before undergoing TPE (*p* < 0.0001; Figure 1A). These data further reflect that TPE was employed as second-line treatment according to existing recommendation in cases of MS attack. Based on this perspective, HDIVMP remains the first-line therapy for MS relapses, and TPE should be reserved for the refractory cases in patients with a relapsing–remitting MS course as previously stated [34]. However, in our study, TPE was effective in 80% of patients with MS, and this finding was concordant with existing data [35,36]. Particularly, we documented a significant reduction in the mRS score after TPE (*p* < 0.0001) as well as a marked EDSS reduction in these patients after TPE (Figure 2B). Although the mechanism of action of TPE is not specific and MS is not an antibody-mediated disease, the high efficacy of TPE in MS may be explained by the removal of pro-inflammatory cytokines and complement components from the bloodstream, resulting in reduced demyelination. Consistent with this view, some authors found that TPE was effective in all the MS patients with immunopathological pattern II characterized by immunoglobulin deposition and complement activation with antibody-complement associated demyelination [37]. In that study, TPE was not effective in other immunopathological patterns or in patients with progressive MS.

### 4.2. TPE in NMOSD/MOGAD and ADEM

Patients with NMOSD/MOGAD and ADEM commonly received HDIVMP before TPE (Figure 1). As consequence, TPE was performed in these patients due to a steroid-refractory course. However, in our study, TPE was ineffective in patients with seronegative NMOSD and MOGAD, while it did not have a relevant impact on ADEM (Figure 2A). A previous study reported that the use of TPE as first-line therapy in NMOSD attack was superior compared to HDIVMP; however, the efficacy of TPE was limited to spinal attack without any advantage over steroids in the case of other CNS manifestations [38]. Moreover, it has been also shown that the effectiveness of TPE in patients with NMOSD was higher if TPE was initiated immediately and fell to 0–1% when TPE was delayed to day 20 even with prior HDIVMP administration [39]. The rationale for the use of TPE in NMOSD and MOGAD is the removal of pathogenetic antibodies (i.e., aquaporin-4 immunoglobulin G and myelin oligodendrocyte glycoprotein IgG antibodies) that are directly associated with the demyelinating attack [40]. Thus, there are several potential reasons for TPE ineffectiveness in our patients. First, the patient with NMO tested negative for serum antibodies. Second, the TPE initiation was delayed both in NMO and MOGAD participants. Third, only the patient with seronegative NMOSD had spinal attack, while the MOGAD patient had supratentorial CNS involvement, thus limiting TPE efficacy. However, we enrolled only two participants with NMOSDs, and these data should be cautiously interpreted.

### 4.3. TPE in MG

TPE reached 100% of effectiveness in the patients with MG crisis with a marked reduction in the MG-ADL score in all the MG patients after TPE (*p* = 0.009; Figure 2C). In our cohort, approximately 55.5% of MG patients were refractory to IVIG and then underwent TPE. Notably, only one MG patient tested positive for MuSK antibodies, while the majority of MG patients tested positive for AChR antibodies that mainly belong to IgG 1 and 3 classes [41]. A recent study showed that the levels of immunoglobulin decreased by 85% after five TPE sessions, and IgG1 and IgG3 antibodies levels remained depleted over a 4-month follow-up [42]. These data are consistent with a rapid and sustained depletion of pathogenetic AChR antibodies in MG, thus explaining the marked reduction in MG-ADL observed in our study.

### 4.4. TPE in Acquired Polyneuropathies

We found a significant mRS score reduction in patients with GBS after TPE (*p* = 0.046; Figure 2A), but this was not true for patients with CIDP and PNSs (all *p* > 0.05; Figure 2A). When considering the MRC sum score, both GBS and CIDP patients showed a slightly increasing muscle strength without reaching statistical significance (Figure 2D). However, TPE was started later in patients with CIDP (Table 3), and they underwent a higher number of TPE cycles since this was employed as maintenance therapy in these patients due to the ineffectiveness of first-line therapies. The effectiveness of short-term TPE in patients with CIDP has been well demonstrated; however, two-thirds of patients often require maintenance TPE [43]. The rationale for the use of TPE in these patients involves preventing the deposition of immunoglobulin and complement on nerve fibers; however, pathogenetic antibodies are not seen in CIDP. Conversely, in patients with paraprotein neuropathies such as anti-MAG, TPE was more effective in those with IgA- and IgG-associated disease rather than IgM paraprotein disease [5]. However, we enrolled only one patient with anti-MAG neuropathy, and we could not provide reliable data on the effectiveness of TPE in this case. On the other hand, it is known that circulating and pathogenetic antibodies directed to gangliosides in GBS induce complement fixation and subsequently peripheral nerve damage [44]. Similar to that noted for MG, the rationale of the use of TPE in patients with GBS involves the rapid depletion of circulating pathogenetic antibodies, resulting in a rapid improvement in the mRS score in our study. However, the discordance between the significant mRS score reduction and the insignificant MRC increase in patients with GBS may be due to the interoperator variability of neurological examination as well as to the fact that MRC may be inappropriate in some cases (e.g., GBS with predominant sensory impairment). Furthermore, it has been shown that TPE is highly effective in axonal form of GBS and less in demyelinating ones [9]. Due to the lower number of GBS patients, we could not conduct a subgroup analysis testing whether TPE effectiveness was different between axonal and demyelinating GBS variants. However, TPE should be initiated as soon as possible in patients with GBS as first-line therapy also considering that the superior of the combination therapy with TPE followed by IVIg is not demonstrated [7,45]. Finally, TPE in patients with PNS, including POEMS, cerebellar degeneration associated with Yo and Hu antibodies, anti-titin myasthenic syndrome, and seronegative PN neuropathy was ineffective (Figure 2A) and almost employed before the obtaining a definite diagnosis. Indeed, most of these patients were initially misdiagnosed as having CIDP. This resulted in a lower than expected effectiveness of TPE in these patients previously diagnosed as having CIDP. Of note, the misdiagnosis of a chronic neuropathy with CIDP is a leading cause of a lower than expected ineffectiveness of TPE [46]. Moreover, it has been shown that antibodies against intracellular antigens such as Hu and Yo are surrogate markers of other underlying diseases, and they are not pathogenetic since neuronal injury in this case is mainly attributed to cytotoxic T cells [47]. In these cases, the rationale of employing TPE is not met. Based on our results, it should be avoided in the case of seronegative or anti-intracellular antigen-associated PNSs.

### 4.5. TPE in Stiff Person Syndrome

Since SPS is a rare disease, we enrolled only one patient. This patient did not undergo to other therapies before TPE, which served as the rescue as well as the maintenance therapy. Although we noted a one-point reduction in the mRS score in this patient, our data are not sufficient to provide reliable data on the use of TPE in SPS. However, globally few data are available in patients with SPS, and TPE seems a good treatment option both for acute exacerbation and long-term maintenance [48].

### 4.6. Predictors of TPE Effectiveness

TPE represented the first-line therapy in 32.2% of patients, especially in those with GBS (55.6%) and MG crisis (44.5%) and less commonly in other patients. IVIg preceded TPE in 27.1% of cases, especially in patients with CIDP and PNSs (*p* < 0.0001; Figure 1B). However, HDIVMP was administered before TPE in 26 (44.1%) participants, mainly with MS, NMOSD/MOGAD, and ADEM (Figure 1A). Notably, the median time from the last IVIg administration and TPE initiation was 41.5 days. Although the sequence from IVIg to TPE is not recommended due to the clearance of circulating immunoglobulin achieved using TPE, in our study, the time between IVIG administration and TPE appeared long enough to avoid Ig removal. These results are in agreement with the existing indications that recommend TPE as second-line therapy in patients with steroid-refractory MS or NMOSD/MOGAD, and the guidelines recommend the use of TPE as the first-line therapy in patients with GBS and MG crisis [6,49]. We conducted a multivariate analysis to explore the factors associated with TPE effectiveness, including the use of TPE as first-line therapy despite the diagnosis. Accordingly, the multivariate analysis showed that the younger age at TPE initiation (aOR = 4.5; *p* = 0.054; Table 3) as well as the use of TPE as first-line therapy (aOR = 5.5; *p* = 0.056; Table 3) were positively associated with highest probability of TPE success. These data did not reach statistical significance probably due to the lower number of individuals included in this study; however, our results further suggest the use of TPE as first-line therapy in patients with neuroimmunological disorders, especially in younger subjects. These data may indicate that TPE should be considered as first-line rescue therapy in patients with MG and GBS. On the other hand, these data may also support the use of TPE as first-line therapy even in patients with MS attack given both the high effectiveness of TPE as well as the younger age of these patients. Finally, sex, number of TPE cycles, and baseline mRS score were not associated with its effectiveness (Table 4).

### 4.7. TPE before Obtaining a Definite Diagnosis

Furthermore, the effectiveness of TPE in patients who underwent this procedure before receiving a definite diagnosis was significantly lower compared to those with a definite diagnosis (50% vs. 80%; *p* = 0.04). At the same time, patients without a definite diagnosis more commonly underwent other treatments before starting TPE (Figure 1). These results reflect, in part, a lower than expected effectiveness of TPE when the diagnosis is not well established. Indeed, the majority of patients who underwent TPE before obtaining a definite diagnosis were subsequently diagnosed with seronegative NMOSD, ADEM, and PNSs. Accordingly, TPE was not effective in these patients in our study (Figure 2). Finally, after the multivariate analysis, the presence of circulating antibodies was identified as the only statistically significant factor associated with TPE effectiveness (aOR = 6.5; Table 4). This is in agreement with the therapeutic rationale of TPE (i.e., removal of pathogenetic circulating antibodies), and it may also explain the greatest effectiveness of TPE in patients with MG in this study.

### 4.8. TPE Safety

In the second part of this study, we explored the frequency of the AEs that occurred during TPE and their associated factors. Overall, the 54.2% of participants reported at least one AE. The total number of AEs was 66 out of 447 TPE sessions with a frequency of 14.8% and an incidence of 1.46 AEs throughout ten sessions. No differences were noted based on sex, age, circulating antibodies, and underlying disease. Although a higher number of participants experienced an AE, the frequency of AEs based on the number of TPE sessions was similar to that reported previously [50,51]. Overall, the majority of AEs were mild or moderate, while severe AEs occurred in 10% of patients (Figure 3A); the most common AE was hypotension (Figure 3B), while the most common severe AE was hypocalcemia crisis (Figure 3B). These data are concordant with a recent study [52]. Of note, all the patients included in this study received 5% human albumin and electrolyte solution as replacement after TPE. For this reason, we did not observe allergic reactions, which are common when plasma is used as replacement fluid [9]. After multivariate analyses, performing more than five TPE sessions was strongly associated with the occurrence of an AE, while each point increase in the mRS score was associated with a 49% higher risk of developing AEs (Table 5). This may be explained by the intrinsic frailty of the patients with neurological diseases, and this is particularly true for those with a severe clinical course. Our result is also supported by the finding of a higher frequency of AEs related to TPE in patients with comorbidity [8,13]. However, we conclude that the safety profile of TPE was acceptable since the majority of the observed AEs were mild or moderate.

### 4.9. Limitations

This study is not exempt from limitations. First, the retrospective design may lead to data underestimation as well as attrition bias since some individuals who underwent TPE were lost to follow-up. Second, the number of participants included was low, yielding a less robust statistical analysis that was mainly descriptive. The lower number of participants in this study may explain why some predictors were not statistically significant, whereas the higher value obtained for some predictors revealed their clinical relevance. Third, the selection bias due to the clinical expertise of our clinical center should be noted. This explains the higher number of patients with MS included in this study. We included only one patient with SPS and three patients with ADEM; therefore, we could not provide stronger data on the use of TPE in these patients. Finally, we did not calculate the total volume of plasma exchanged at each TPE session.

## 5. Conclusions

Results from this pilot study indicate that TPE initiation before the obtaining a definite diagnosis resulted in its reduced effectiveness, especially in patients with unexplained chronic polyneuropathies or ADEM, and it should be avoided in patients with an uncertain diagnosis. Moreover, we encourage the use of TPE as first-line rescue therapy and as soon as possible in patients with MG and GBS, especially in younger patients, given the higher effectiveness of TPE in antibodies-mediated neuroimmunological disorders. We also suggest to avoid TPE in diseases mainly sustained by innate or cytotoxic T cell autoimmunity wherein the antibodies are not pathogenetic but an just epiphenomenon of underlying disease, such as that noted in PNSs. Based on the results from our study, we would encourage the use of TPE in younger patients with MS attack, and we also would encourage future studies exploring the role of TPE in the treatment of SPS, CIDP, and NMOSD/MOGAD. Finally, although TPE showed a favorable safety profile, results from this study suggest that care should be taken when TPE is started in frail patients. In addition, the minimum effective number of cycles should be performed to minimize AE occurrence. Finally, we further encourage the early and prompt management of frail patients with higher disease severity who underwent TPE in order to reduce the frequency and the magnitude of AEs in these patients.

## Figures and Tables

**Table 1:** Clinical indications to perform therapeutic plasma exchange.

TPE Indication	*n* (%)
Definite diagnosis	45 (76.3)
MS attack	20 (44.4)
MG crisis	9 (20)
GBS	9 (20)
CIDP	3 (6.6)
PNSs	2 (4.4)
MOGAD	1 (2.2)
SPS	1 (2.2)
Diagnosis unknown	14 (23.7)
Acute myelitis or encephalomyelitis	9 (64.3)
Subacute or chronic polyneuropathy	5 (35.7)

Abbreviations: TPE, therapeutic plasma exchange; MS, multiple sclerosis; MG, myasthenia gravis; GBS, Guillain–Barré syndrome; CIDP, chronic inflammatory demyelinating polyneuropathy; PNSs, paraneoplastic syndromes; MOGAD, myelin oligodendrocyte glycoprotein antibody-associated disease; SPS, stiff-person syndrome.

**Table 2:** Demographic and clinical features of the participants included in this study.

Parameters	
Women, *n* (%)	36 (61)
Age at disease onset, y, median (IQR)	43 (30–62)
Age at diagnosis, y, median (IQR)	45 (31–64)
TPE as first-line rescue therapy, *n* (%)	19 (32.2)
Age at TPE initiation y, median (IQR)	52 (41–65)
Time from acute symptom to TPE, d, median (IQR)	26 (14–60)
Number of TPE cycles, median (IQR)	5 (5–9)
At least two HA, *n* (%)	13 (22)
Number of HA, mean ± SD	1.39 ± 1
Number of TPE cycles per HA (95% CI)	5.4 (4.1–7.1)
Positive testing for serum antibodies, *n* (%)	17 (28.8)
AChR	8 (47)
MuSK	1 (5.9)
Antigangliosides	2 (11.8)
Hu	1 (5.9)
Yo	1 (5.9)
MAG	1 (5.9)
MOG	1 (5.9)
Titin	1 (5.9)
GAD	1 (5.9)

Abbreviations: IQR, interquartile range; TPE, plasma exchange; HDIVMP, high-dose intravenous methylprednisolone; IVIG, intravenous immunoglobulin; HA, hospital admission; AChR, acetylcholine receptor; MuSK, muscle kinase; MAG, myelin-associated glycoprotein; MOG, myelin-oligodendrocyte glycoprotein; GAD, glutamic acid decarboxylase.

**Table 3:** Comparison of clinical features in study participants according to final diagnosis.

Diagnosis	Female *n* (%)	Age at TPE, y	Days to TPE	No. TPE Cycles
MS	18/25 (72)	42 (31–47)	46 (14–75)	5 (5–6)
MG	7/9 (77.8)	63 (52–69)	25 (8–41)	6 (4–15)
GBS	3/9 (33.3)	65 (59–67)	17 (15–21)	5 (5–10)
CIDP	1/4 (20)	69 (64–70)	51 (22–151)	16 (5–33)
PNSs	3/5 (60)	46 (38–72)	26 (26–94)	5 (5–5)
ADEM	1/3 (33.3)	54 (49–60)	29 (22–43)	5 (4–6)
NMOSD/MOGAD	2/2 (100)	35 (26–44)	17 (15–18)	8 (7–10)
*p*	0.09 *	<0.0001	0.09	0.35

* p for trend. Data are reported as median and interquartile range within round brackets. MS, multiple sclerosis; MG, myasthenia gravis; GBS, Guillain–Barré syndrome; CIDP, chronic inflammatory demyelinating polyneuropathy; PNS, paraneoplastic syndrome; ADEM, acute disseminated encephalomyelitis; NMOSD, neuromyelitis optica spectrum disorder; MOGAD, myelin oligodendrocyte glycoprotein antibody-associated disease.

**Table 4:** Multivariate logistic regression analysis exploring the predictors associated with plasma exchange effectiveness.

Predictors	B	aOR	95% CI for aOR		*p*
Male	−0.15	0.86	0.22	3.4	0.83
Age < 50 y at TPE	1.51	4.5	0.97	20.9	0.054
TPE as first-line therapy	1.70	5.5	0.96	31.6	0.056
Positive serum antibody	1.87	6.5	1.01	40.1	0.045
≥5 TPE sessions	0.65	1.6	0.37	6.6	0.54
mRS score before TPE	0.39	1.48	0.70	3.20	0.31

aOR, adjusted odds ratio; CI, confidence intervals; TPE, plasma exchange; mRS, modified Ranking Scale.

**Table 5:** Multivariate logistic regression analysis exploring the predictors associated with the occurrence of adverse event following plasma exchange.

Predictors	B	aOR	95% CI for aOR		*p*
Male	0.31	1.36	0.4	4.65	0.62
Age < 50 y at TPE	−0.17	0.85	0.26	2.76	0.78
TPE as first-line therapy	0.26	1.29	0.37	4.47	0.69
≥5 TPE sessions	1.76	5.79	1.67	20.1	0.006
mRS score before TPE	−0.68	0.51	0.25	1.04	0.063

aOR, adjusted odds ratio; CI, confidence intervals; TPE, plasma exchange; mRS, modified Ranking Scale.

## Data Availability

Data supporting this research are available upon request from any qualified investigator to the corresponding author.
